# Salusin-β as a powerful endogenous antidipsogenic neuropeptide

**DOI:** 10.1038/srep20988

**Published:** 2016-02-12

**Authors:** Noriko Suzuki-Kemuriyama, Tae Nakano-Tateno, Yuji Tani, Yukio Hirata, Masayoshi Shichiri

**Affiliations:** 1 Department of Endocrinology, Diabetes and Metabolism, Kitasato University School of Medicine, Kanagawa 252-0374, Japan; 2 Tokyo Medical and Dental University Graduate School, Tokyo 113-8519, Japan; 3 Institute of Biomedical Research and Innovation Hospital, Hyogo, Japan

## Abstract

Salusin-β is an endogenous parasympathomimetic peptide, predominantly localized to the hypothalamus and posterior pituitary. Subcutaneously administered salusin-β (50 nmol/mouse) significantly increased water intake but did not affect locomotor activity or food intake. The salusin-β-induced increase in water intake was completely abrogated by pretreatment with muscarinic antagonist, atropine sulphate. In contrast, intracerebroventricular injection of salusin-β, at lower doses (10–100 fmol/mouse) caused a long-lasting decrease in water intake and locomotor activity throughout the entire dark phase of the diurnal cycle. Pre-injection of intracerebroventricular anti-salusin-β IgG completely abrogated the central salusin-β mediated suppression of water intake and locomotor activity. These results demonstrate contrasting actions of salusin-β in the control of water intake via the central and peripheral systems and highlight it as a potent endogenous antidipsogenic neuropeptide.

Salusin-β is an endogenous parasympathomimetic peptide with 20 amino acid residues and was originally identified using bioinformatic analyses^1^. Intravenous injection of salusin-β causes profound decreases in blood pressure and heart rate of rats and these acute systemic effects can be completely blocked by pretreatment with antimuscarinic agent, atropine sulphate^1^,^2^. Salusin-β is expressed ubiquitously throughout human and murine tissues^1^,^3^ and is present in human plasma and urine^4^,^5^,^6^. Plasma total salusin-β levels decrease following parasympathetic nerve stimulation^5^ and elevated levels can be detected in atherosclerotic diseases^4^. Salusin-β is especially abundant in vasopressin-expressing neurons of the hypothalamus and posterior pituitary^3^,^7^ and stimulates vasopressin and oxytocin secretion from the neurohypophysis^1^,^8^. Its expression can be increased by osmotic stimuli and dehydration^8^. Thus, salusin-β released from the posterior pituitary is expected to be involved in the regulation of fluid homeostasis as a neuropeptide. In the local vasculature, salusin-β stimulates the progression of atherosclerotic lesions by accelerating macrophage foam cell formation^9^,^10^, enhancing proinflammatory molecule expression and increasing the adhesion of monocytes to endothelial cells^11^. Furthermore, salusin-β is secreted from human monocytes/macrophages^12^ and acts to regulate cell proliferation and apoptosis^1^,^13^,^14^.

The vagus nerve is the major neuroanatomical link between the peripheral digestive system and the brain, with parasympathetic nerve stimulation able to influence water intake and salivary secretion^15^,^16^. Circulating factors can be mediators of efferent signals for the cephalic phase of digestion but may also act on the vagal afferent pathway to influence appetite^17^. For example, plasma ghrelin levels determine food-induced reward seeking behaviors, whilst peripheral administration of acetylcholine receptor antagonists results in reduced feeding response^18^. Receptors for appetite-modifying peptides, such as cholecystokinin, peptide YY_3–36_ and glucagon-like peptide-1 are expressed on the vagus nerve and their actions are abolished by vagotomy^19^,^20^,^21^. Thus, neuroendocrine peptides may affect feeding and drinking behaviors via the parasympathetic nervous system. However, the reported actions exerted by many bioactive peptides used supraphysiological doses either systemically or intracranially. Previous intracerebroventricular studies^22^,^23^,^24^ used concentrations of peptides that were three orders of magnitude higher than endogenous concentrations^25^,^26^,^27^, with a limited number of neuroendocrine peptides showing any essential endogenous role in the regulation of feeding and drinking behaviors^28^.

More than 80% of daily spontaneous activities occur during the dark phase in rodents^29^, so behavior analysis during the dark period is essential to understand physiological actions of bioactive peptides. However, almost all previous behavioral studies were performed during a limited period after administration of bioactive peptides^22^,^23^,^24^,^30^. Traditional automated monitoring of small animal behaviors is limited by the ability of monitoring apparatus to accurately detect subtle behaviors or food and fluid intake. The current study was designed to determine whether centrally and peripherally administered salusin-β modulated eating, drinking and locomotion during the dark phase of the diurnal cycle. The study utilized a recently developed computerized activity-tracing chamber that is equipped with a fluid intake sensor and an accurate scale for measuring food intake^31^, enabling continuous monitoring of spontaneous animal behaviors throughout the dark phase.

## Results

### Effects of subcutaneous injection of salusin-β on cumulative water intake, locomotor activity and food intake

**Figure 1:**
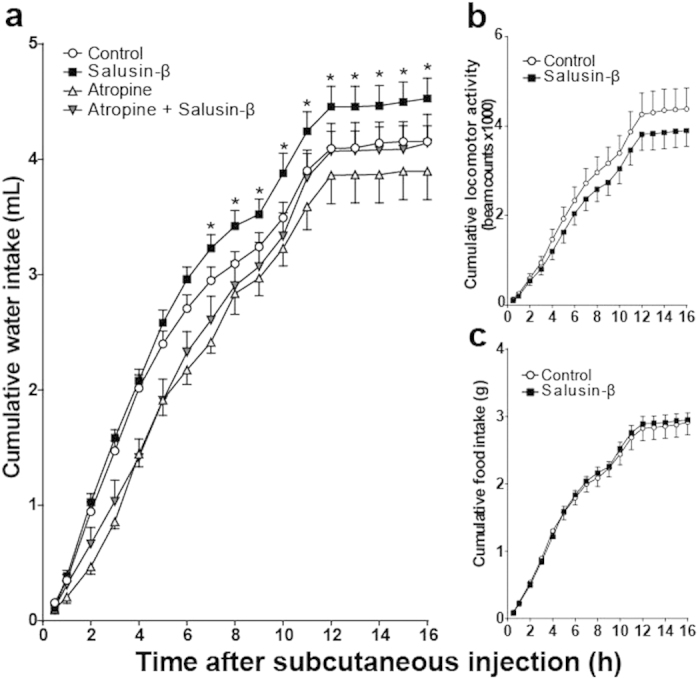
Effects of subcutaneous salusin-β on cumulative water intake, locomotor activity, and food intake. Salusin-β (50 nmol), dissolved in 100 μl 0.1% TFA/0.1% NP-40 saline or 100 μl control 0.1% TFA/0.1% NP-40 saline solution, was subcutaneously injected to *ad libitum* watered and fed mice, 10 min before dark period onset and the cumulative water intake (**a**), spontaneous locomotor activity (**b**) and food intake (**c**) were monitored throughout the entire dark phase of the diurnal cycle. Cumulative water intake, locomotor activity and food intake in salusin-β-treated mice without (closed square, n = 13) or with subcutaneous pre-injection of 3 mg/kg atropine sulphate (closed triangle, n = 9) are compared with mice given the control solution (open circle, n = 13) or with atropine alone (open triangle, n = 9). Data are presented as the mean ± S.E.M. *p < 0.05 vs control.

The effects of subcutaneous salusin-β (50 nmol) on the food/water intake and locomotor activity of conscious mice were investigated alone and after subcutaneous pretreatment with the muscarinic antagonist, atropine sulphate. Mixed-design ANOVA detected a significant interaction between treatment and time (F(48,640) = 1.939, p < 0.0001) on hourly water intake. Cumulative water intake was significantly increased following salusin**-**β administration compared with control animals after 6-h time points (Fig. 1a). This increase lasted throughout the entire dark phase. Atropine injection completely abrogated the salusin**-**β mediated increase in water intake (Fig. 1a). However, subcutaneous salusin-β did not significantly affect locomotor activity (Fig. 1b) or food intake (Fig. 1c). Pretreatment with atropine increased baseline locomotor activity but did not affect food intake (data not shown).

### Effects of intravenous injection of salusin-β on urine volume, sodium and potassium concentrations

**Figure 2:**
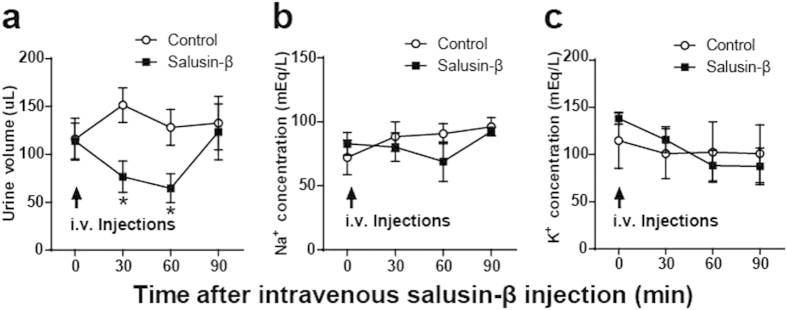
Effects of intravenous salusin-β injection on urinary volume, plasma sodium and potassium concentrations in rats. Salusin-β (1 nmol/kg) dissolved in 100 μl 0.1% TFA/0.1% NP40/0.1% Tween 20 saline (closed square) or 100 μl control 0.1% TFA/0.1% NP40/0.1% Tween 20 saline solution (open circle) was intravenously (i.v.) injected into 5 rats and urine volume (**a**), plasma sodium (**b**) and plasma potassium levels (**c**) were determined before and at 30 min intervals after injection. Data are expressed as mean ± S.E.M. *p < 0.05 compared with control animals.

As salusin-β exerts potent hypotensive effects, we tested whether the salusin-β-induced dipsogenic effect was mediated via diuresis/natriuresis. Changes in urine volume and urinary electrolyte excretion were measured in anesthetized rats, following intravenous salusin-β administration. Salusin-β (1 nmol/kg) caused temporary decreases in urine volume, lasting less than 90 min (Fig. 2a). However, urinary sodium and potassium excretions were unaffected by salusin-β (Fig. 2b,c). These data negate the possibility that salusin-β stimulates water intake as a result of diuresis/natriuresis.

### Effects of intracerebroventricular injection of salusin-β on water intake, locomotor activity and food intake

**Figure 3:**
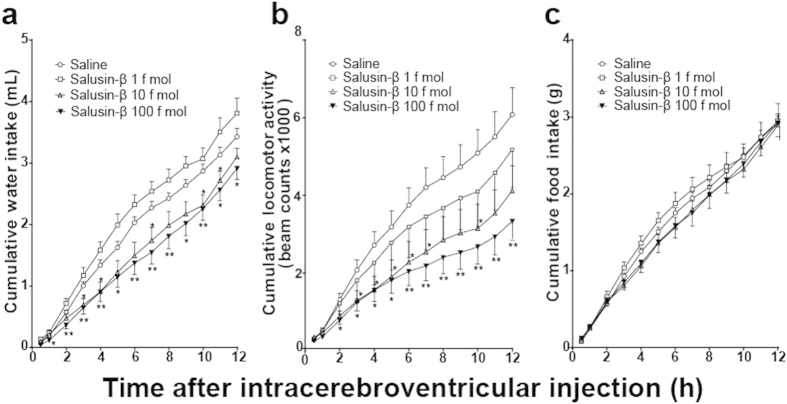
Effects of intracerebroventricular salusin-β on cumulative water intake, locomotor activity and food intake. Salusin-β dissolved in 100 μl 0.1% TFA/0.1% NP40 saline or 100 μl control 0.1% TFA/0.1% NP40 saline solution was injected via an intracerebroventricular catheter to *ad libitum* watered and fed mice, 10 min before dark period onset and the cumulative water intake (**a**), spontaneous locomotor activity (**b**) and food intake (**c**) were measured. Mice injected without (open circle, n = 20) or with 1 fmol (open square, n = 7), 10 fmol (open square, n = 13) or 100 fmol (open square, n = 18) salusin-β were monitored throughout the entire dark phase of the diurnal cycle. Data for locomotor activity, water intake and food intake are expressed as mean ± S.E.M. *p < 0.05 compared with control mice.

**Figure 4:**
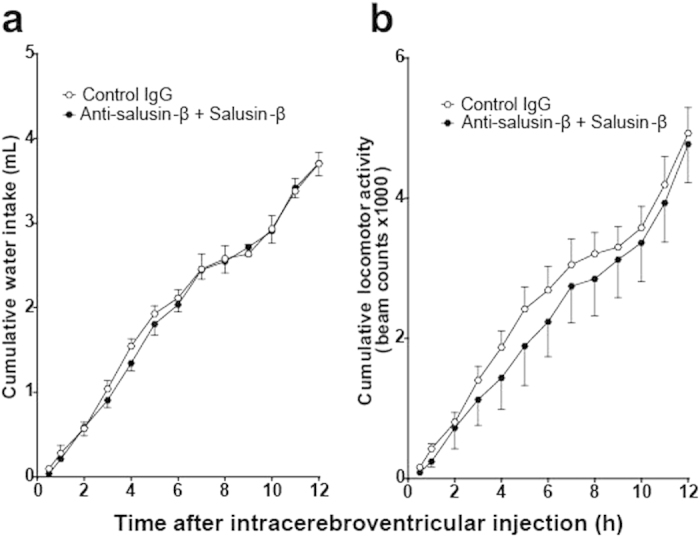
Effects of anti-salusin-β IgG pretreatment on salusin-β-induced suppression of water intake and spontaneous locomotor activity. Anti-salusin-β IgG was preinjected 1 h before intracerebroventricular injection of 0.1% TFA/0.1% NP40 saline containing salusin-β (100 fmol, closed circle, n = 5) or control 0.1% TFA/0.1% NP40 saline solution (open circle, n = 5) via intracerebroventricular catheter and cumulative water intake (**a**) and locomotor activity (**b**) throughout the entire dark phase of the diurnal cycle of mice (with *ad libitum* access to water and food) were monitored. Locomotor activity and water intake data are expressed as mean ± S.E.M.

Intracerebroventricular administration of very low doses of salusin-β to mice, with unlimited access to tap water and chow, produced a sustained reduction in drinking behavior and spontaneous locomotor activity (Fig. 3a,b). The cumulative water intake of mice receiving 10 and 100 fmol salusin-β remained distinctly lower than control animals during the entire 12 h dark phase (Fig. 3a). Salusin-β treatment dose-dependently decreased cumulative locomotor activity from 120 min after administration until the end of dark phase (Fig. 3b) but did not affect cumulative food intake at any time point (Fig. 3c). Pre-injection of intracerebroventricular anti-salusin-β IgG completely abrogated the suppressant effects of intracerebroventricular salusin-β on water intake and locomotor activity (Fig. 4a,b).

**Figure 5:**
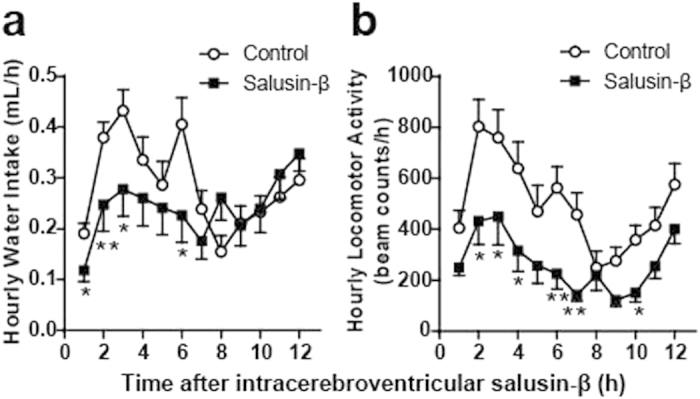
Effects of intracerebroventricular injection of salusin-β on hourly water intake and locomotor activity in the *ad libitum* watered and fed mice. Salusin-β (100 fmol) dissolved in 100 μl 0.1% TFA/0.1% NP40 saline (closed square, n = 18) or 100 μl control 0.1% TFA/0.1% NP40 saline solution (open circle, n = 20) was injected via intracerebroventricular catheter and water intake (**a**) and spontaneous locomotor activity (**b**) data were monitored throughout the entire dark phase of the diurnal cycle. Hourly data for water intake and locomotor activity were calculated using the experiments performed in Fig. 3 and expressed as mean ± S.E.M. *p < 0.05 compared with control mice.

To assess the time-course of salusin-β effects on water intake and locomotor activity, the above behavior data following a single intracerebroventricular injection of vehicle or 100 fmol salusin-β were reanalyzed at hourly intervals up to 12 h. Mixed-design ANOVA revealed a significant interaction of treatment and time (F(12,432) = 2.184, p = 0.012) on water intake, induced by 100 fmol salusin-β. Significant effects of treatment (F(1,36) = 5.886, p = 0.002) and time (F(12,432) = 8.286, p < 0.0001) were also detected. Mann–Whitney U *post hoc* tests showed that, following salusin-β treatment, water intake was significantly inhibited at 0–1 h, 1–2 h, 2–3 h and 5–6 h compared with vehicle-treated mice (Fig. 5a). The antidipsogenic effect disappeared 7 h after injection and was not followed by rebound water intake to compensate for the reduction (Fig. 5a). There was a significant interaction effect between treatment and time (F(36,648) = 3.688, p < 0.0001) on locomotor activity following administration of 100 fmol salusin-β (Fig. 5b). The decrease in locomotor activity was also evident in the second hour after intracerebroventricular injection of salusin-β and was present intermittently over 10 h (Fig. 5b).

### Salusin-β did not change plasma osmolality, plasma arginine vasopressin (AVP) or urinary AVP levels

**Figure 6:**
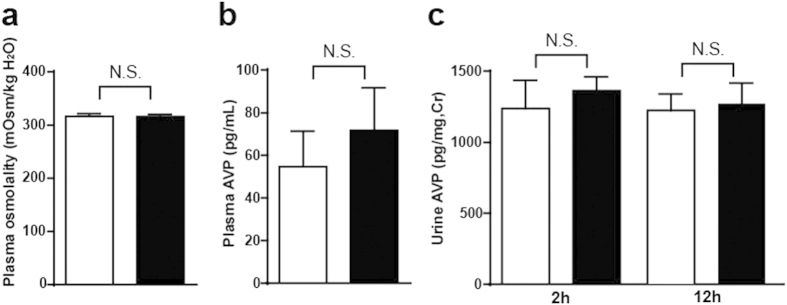
Effects of intracerebroventricular injection of salusin-β on plasma osmolality and plasma and urine AVP levels. Plasma samples obtained 2 h after intracerebroventricular injection of salusin-β (closed column, n = 5) or control saline (open column, n = 5) were used to determine osmolality (**a**) and arginine vasopressin (**b**). Urine was collected in the metabolic cages 2 h and 12 h (**c**) after intracerebroventricular salusin-β (closed column, n = 6) or saline (open column, n = 6) to measure the arginine vasopressin concentrations.

Plasma osmolality and plasma AVP levels were determined 2 h after intracerebroventricular injection of salusin-β to coincide with the peak suppressant effects on water intake and locomotor activity. However, they did not show any difference from pretreatment levels (Fig. 6a,b). Urinary AVP levels in the urine collected at 2 h and 12 h following intracerebroventricular salusin-β injection were also unchanged (Fig. 6c).

### Qualitative changes in animal behavior

In order to assess whether intracerebroventricular salusin-β caused any other observable changes in general animal behaviors, we watched video recordings of the entire dark phase of the diurnal cycle. Mice studied during the dark phase often exhibited more exploratory behavior than in the light phase. However, there were no changes in the amount of jumping or rearing observed in any group and no significant differences observed between the control and salusin-β-treated mice (data not shown).

## Discussion

The present study provides the first evidence that peripherally administered salusin-β stimulates water intake, whereas centrally administered salusin-β causes a very potent suppressant effect on both spontaneous water intake and locomotor activity. Subcutaneous injection of salusin-β increased water intake, but did not significantly change locomotor activity or food intake. The stimulatory effects of subcutaneously administered salusin-β on water intake were inhibited by subcutaneous pre-injection of atropine. We have previously reported that intravenous administration of salusin-β induced hypotension and bradycardia in rats and that these effects were completely blocked by pretreatment with atropine^2^. There is evidence suggesting that acetylcholine activates muscarinic receptors to induce drinking. For example, systemic administration of pilocarpine, a muscarinic receptor agonist, induces water intake^32^, whilst peripheral administration of atropine blocks the dipsogenic effect of central administration of acetylcholine, carbachol, and other acetylcholine agonists^33^,^34^,^35^. We also examined whether salusin-β exerted any diuretic actions. Intravenous injection of salusin-β to anaesthetized rats did not induce diuresis but did decrease urine excretion. This may be a result of reduced renal blood flow caused by the temporary hypotensive and bradycardic effects of salusin-β^1^,^2^. Taken together, these results suggest that the stimulation of water intake by subcutaneous injection of salusin-β is mediated through a cholinergic mechanism.

In the current study, increased water intake following subcutaneous injection of salusin-β occurred relatively slowly, in comparison to many other dipsogenic reagents in the literature. Salusin-β, once in the peripheral circulation, is likely to be bound by plasma proteins^36^. A significant amount of salusin-β in the peripheral circulation may not exist as a free peptide, so is unable to exert rapid biological effects^36^. Plasma levels of salusin-β from humans show diurnal changes, which mirror parasympathetic nervous activity^4^. Stimulation of parasympathetic nervous activity lowers plasma salusin-β levels but this response is also delayed^5^. In contrast, salusin-β administered intravenously, as a free peptide, exerts rapid but temporary haemodynamic actions. In the present study, the decrease in urine volume following intravenous salusin-β disappeared within an hour, while the sodium and potassium excretion rate was unaltered. It is predicted that salusin-β molecules in the peripheral circulation would regulate water intake and haemodynamics via cholinergic mechanisms. However, the biological effects of salusin-β on body fluids may be modulated through binding to plasma proteins, which results in more modest effects over longer periods of time.

In contrast to subcutaneously administered salusin-β, intracerebroventricular injection dose-dependently decreased water intake and locomotor activity of *ad libitum* watered and fed mice. We found that reduced drinking behavior occurred at a salusin-β dose three orders of magnitude lower than that of other well-described humoral factors. The central suppressant effect of salusin-β on water intake was elicited at 10–100 fmol/mouse, whereas changes in drinking behavior induced by intracerebroventricular administration of angiotensin II and atrial natriuretic peptide generally required ~100 pmol/mouse and ~1 nmol/rat, respectively^22^,^37^. A decrease in cumulative water intake was detected within 1 h of intracerebroventricular salusin-β administration, which lasted throughout the 12 h observation period. Our hourly analysis revealed that reduced water intake was detected from 1 to 2 h, up to 6 h. In the latter half of the dark phase, drinking behavior was similar in salusin-β and saline vehicle groups. These data suggest that salusin-β reduced water intake during the early hours of the dark phase, which was when the highest drinking activity was observed in saline vehicle mice.

Others and we have previously reported that salusin-β stimulates the secretion of vasopressin and oxytocin from the neurohypophysis^1^,^8^. The salusin-β doses of 10–100 nM required *in vitro* to stimulate vasopressin and oxytocin release were relatively high^8^. However, the potency of salusin-β is markedly reduced by dissolving synthetic salusin-β without nonionic detergents and then aliquoting into plastic tubes^8^,^38^. Highly purified salusin-β peptides quickly and tightly adhere to the surface of polypropylene and polystyrene when dissolved in aqueous solution. This adsorption can be prevented only by the addition of low concentrations of nonionic detergents, such as NP-40 and tween 20^6^,^36^,^39^. We tested whether the observed antidiuresis after intracerebroventricular injection of low doses of salusin-β was due to increased vasopressin secretion. Intracerebroventricular administration of 10–100 fmol salusin-β, which corresponds to 0.1–1 nM in the mouse cerebroventricle, was not sufficient to modulate plasma vasopressin levels or osmolality at 2 h after treatment, when water intake and locomotor activity showed maximal suppression. Taken together, our results suggest that salusin-β-induced antidiuresis was not mediated through the stimulation of vasopressin release.

We also show for the first time that low doses of salusin-β potently decreased spontaneous locomotor activity. A dose-dependent decrease in cumulative locomotor activity was detected 2 h after treatment and lasted throughout the 12 h observation period. Hourly analysis revealed a tendency for reduced locomotion, particularly during the early stages but continuing throughout the entire dark phase. The monitoring system used in the current study allowed us to detect spontaneous locomotor activity over the entire dark phase by counting photo-beam interruptions. However, these beam interruptions register only horizontal movements and do not account for jumping or rearing activity. Qualitative behavioral changes were recorded using a high-sensitivity, digital video camera. No obvious changes in behaviors such as jumping or rearing were observed in either control or treated animals, so central administration of salusin-β decreased overall spontaneous physical activity but without affecting any vertical physical activity.

As intracerebroventricular injection of salusin-β reduced locomotor activity, salusin-β-treated animals may have made fewer approaches to the water bottle. Most species have a close relationship between drinking and feeding behaviors^40^,^41^ and approximately 80% of spontaneous water intake is temporally associated with feeding in rats^42^. However, cumulative food consumption after salusin-β injection accurately measured by the monitoring device used in this study was unaffected at all time points, which is clearly distinct from the potent, long-lasting decreases on water intake and locomotor activity. Since anti-salusin-β IgG abolished the reduction in both water intake and locomotor activity, we conclude that salusin-β may be a potent endogenous suppressor of both water intake and locomotor activity.

In the present study, subcutaneous injection of salusin-β caused an increase in water intake, while intracerebroventricular injection markedly suppressed water intake and locomotion. This paradoxical effect is likely due to salusin-β actions at central and peripheral sites that independently regulate drinking behavior. Salusin receptors have not yet been identified but salusin-β has been shown to bind and activate mouse mas-related G protein-coupled receptors (MrgA1)^43^, which are involved in modulating nociception^44^. In rats, salusin-β-like immunoreactivity is abundant in the hypothalamus and pituitary and is also detectable in immune and gastrointestinal tissues^3^. However, it is not certain whether salusin-β acts mainly in the hypothalamus or in the area postrema or other regions. Some other peptides also exhibit paradoxical effects after central versus peripheral administration. For example, insulin injected intravenously stimulates appetite by inducing hypoglycaemia but when injected into the brain it reduces feeding behaviors^45^. It has also been reported that central infusion of atrial natriuretic factor in conscious rats inhibits basal vasopressin release^46^,^47^, whereas intravenous infusion reduces dehydration and hemorrhage-induced vasopressin release^48^. These findings further support the hypothesis of a role for neuroendocrine peptides in the central control of body fluid balance and electrolyte regulation.

In summary, peripheral administration of salusin-β increased water intake through a cholinergic mechanism, whereas central administration of lower doses potently reduced water intake and locomotor activity. Although the central mechanisms of salusin-β-induced changes in water intake and locomotion remain elusive, a role for salusin-β as a potent endogenous regulator of spontaneous water intake and locomotor activity has been demonstrated.

## Methods

### Animals

Adult male C57BL/6J mice weighing 20–25 g (CLEA Japan, Tokyo, Japan) and adult male Sprague–Dawley rats weighing 250–300 g (Charles River Japan, Shiga, Japan) were maintained under controlled temperatures (23–25 °C), on a 12-h light-dark cycle (lights on 07:00 to 19:00), with free access to food (standard laboratory powder chow; MF, Oriental Yeast, Tokyo, Japan) and water. All animal experimental procedures were approved by the Animal Experimentation and Ethics Committee of the Tokyo Medical and Dental University and by the Kitasato University School of Medicine. Procedures were performed in accordance with either the Tokyo Medical and Dental University Guideline for the Care and Use of Experimental Animals or the guidelines for animal experiments by Kitasato University School of Medicine.

### Implantation of cannulas in the cerebroventricle

Mice were anesthetized with sodium pentobarbital (60 mg/kg i.p.) and stereotaxically implanted with a stainless steel guide cannula (EKC-0502A, BioResearch Center, Nagoya, Japan) in the lateral ventricle as previously described^31^. The stereotaxic coordinates for the lateral ventricle were AP 0.5 (0.5 mm posterior to Bregma), L1 (1 mm left from mid-sagittal line) and H2.0 (2.0 mm below Bregma) and the cannula was secured to the skull using stainless steel screws and dental acrylic cement (GC UNIFAST II, GC Corporation, Tokyo, Japan). After the procedure, a stainless wire stylet was inserted into the cannula to prevent coagulation. The mice had one week of postoperative recovery, after which they were handled daily to equilibrate their arousal levels.

### Subcutaneous and intracerebroventricular injection

Subcutaneous injection was carried out without anesthesia just before the start of the dark period. After two days of habituation with saline injections mice were subcutaneously injected with 50 nmol/mouse of human salusin-β (Peptide Institute Inc., Osaka, Japan) dissolved in 100 μl solvent (96%, propylene glycol, 4% ddH_2_O) or with 100 μl of solvent alone using a 25 G needle. Atropine sulphate (Sigma, St. Louis, MO) was administrated subcutaneously, 3 mg/kg, 30 min before salusin-β administration.

For intracerebroventricular administration, unanaesthetized mice were injected manually with 1 μl of 0.1% TFA/0.1% NP-40 saline solution alone or with 1.0, 10, or 100 fmol salusin-β over 1 min via the indwelling cannula connected to a Hamilton syringe. Intracerebroventricular injections were performed within 10 min prior to the dark period. For immune neutralization studies, mice received an intracerebroventricular injection of either rabbit anti-salusin-β IgG (0.01 μg) or control IgG (0.01 μg)^3^,^7^, 1 h before salusin-β administration. After the completion of experiments, animals were sacrificed and blue dye (5% glycerin, 0.05% bromophenol blue, 0.05% xylene cyanol) was injected through the cannulae. The brains were then sectioned and cannula placement was verified. A flow of cerebrospinal fluid dyed blue from the lateral ventricle to third ventral ventricle was considered correct cannula placement. Mice with cannulae not placed in lateral ventricle were excluded from the analysis.

### Measurement of spontaneous activity and water/food intake

The locomotor activity, water and food intake were recorded using a simultaneous monitoring system (ACTIMO-100 M combined with MFD-100, Shinfactory, Fukuoka, Japan) as described previously 31. The monitoring system uses beam sensor technology that allows comparisons between individual animals with a high degree of precision compared with electrostatic and body temperature sensor systems. Sensors were located every 2 cm along the floor of the enclosure and detected animal movements with an infrared beam every 0.5 s. Simultaneous interruptions of more than two neighboring beams were recorded as “an activity output” by ACTIMO-DATA software (Shinfactory, Fukuoka, Japan) to eliminate artifacts elicited by respiration or nose/tail movements. Water intake was measured by a drop counting system, which utilizes a water bottle attached to a transfusion kit (TERMO, Tokyo, Japan), with each drop of water constituting 17 μl recorded as a signal. A food container filled with standard powder chow was placed lower than the floor, forcing the mice to crawl through an opening to reach it. The width of the feeding adapter was adjusted to fit the size of each mouse in units of 1 mm, to prevent mice from dragging food into their bedding and avoid spillover. The minimum quantity of measurable food was 0.01 g using the microbalance. Movement signal counts were imported in real-time using the Spike2 analysis program (Cambridge Electronic Design, Cambridge, UK), while water and food intake were recorded simultaneously every 3 minutes. Mice were placed in the individual chambers and housed in these cages for 3 days to familiarize them with the recording environment. The experiments were performed during the dark phase (19:00 to 07:00) in a room that was completely isolated from external noises.

### Bioassay procedure for detection of diuretic effects

Male Sprague–Dawley rats were anesthetized with sodium pentobarbital (60 mg/kg i.p.) and their urethras ligated as previously described^49^. Urine was collected from the urinary bladder using a 27 G needle and the wound closed with Vetbond (3 M Company, St. Paul, MN). The volume of urine collected at 30 min intervals for 120 min was measured before and after intravenous administration of salusin-β (1.0 nmol/kg), dissolved in 0.4%TFA, 0.1% NP-40, or saline in 0.4%TFA, 0.1% NP-40. Sodium and potassium content was measured using the Abaxis VetScan VS2 chemistry analyzer with the Comprehensive Diagnostic Profile reagent rotor (Abaxis Inc., Union City, CA) as described previously^50^.

### Plasma and urine measurements

Mice were acclimated to the individual metabolic chambers for 3 days before receiving intracerebroventricular injections of 100 fmol salusin-β or saline at beginning of dark period and then placed back in the chambers. Urine excretion was determined using volumetric measurements and 2 h after injection mice were decapitated without anesthesia and blood samples were collected as described previously^51^,^52^. Plasma Arg8-AVP was measured using the Mitsubishi Chemical laboratory service (Tokyo, Japan). The osmolality of plasma and urine was estimated using a freezing point osmometer (Fiske Osmometer Model 110: Fiske Associates, Norwood, MA).

### Video observation

To assess qualitative changes in animal behavior, the nocturnal activity of four pairs of mice was monitored. After intracerebroventricular injection of saline and salusin-β, behavior was recorded by an ultra low light high resolution CCD Video camera (Watec WAT-232) equipped with a recorder (DV-AC 82, SHARP). The total nocturnal mobility time of individual animals was recorded for 12 h (from 19.00 to 07.00). The minimum illumination of the video camera was 0.006 lux, F1.2, the effective pixels were 38 × 104, and the resolution was 540 TV lines.

### Statistical analysis

Values are expressed as the mean ± S.E.M. Differences among groups in food/water intake and locomotor activity were analysed using SPSS Statistics (IBM Corporation, Somers, NY) software, version 22.0 to perform mixed-design ANOVAs, with treatment as the between subjects factor and time as the within subjects factor^53^. *Post hoc* comparisons were performed using Mann–Whitney U tests. All other data were analysed using Student’s *t* test, Wilcoxon’s test or Mann–Whitney U test, as appropriate. A value of p < 0.05 was considered statistically significant.

## Additional Information

**How to cite this article**: Suzuki-Kemuriyama, N. *et al.* Salusin-β as a powerful endogenous antidipsogenic neuropeptide. *Sci. Rep.* **6**, 20988; doi: 10.1038/srep20988 (2016).
